# Active methods of mercury removal from flue gases

**DOI:** 10.1007/s11356-018-1772-1

**Published:** 2018-03-23

**Authors:** Marta Marczak, Stanisław Budzyń, Jakub Szczurowski, Krzysztof Kogut, Piotr Burmistrz

**Affiliations:** 1 Faculty of Energy and Fuels, AGH University of Science and Technology, Mickiewicz Avenue 30, 30-059 Krakow, Poland; 2 AGH University of Science and Technology, AGH Centre of Energy, Czarnowiejska 36, 30-054 Krakow, Poland

**Keywords:** Solid fuels, Sub-bituminous coal, Lignite, Anthropogenic emission, Mercury removal, Flue gases purification, Sorbents

## Abstract

Due to its adverse impact on health, as well as its global distribution, long atmospheric lifetime and propensity for deposition in the aquatic environment and in living tissue, the US Environmental Protection Agency (US EPA) has classified mercury and its compounds as a severe air quality threat. Such widespread presence of mercury in the environment originates from both natural and anthropogenic sources. Global anthropogenic emission of mercury is evaluated at 2000 Mg year^−1^. According to the National Centre for Emissions Management (Pol. KOBiZE) report for 2014, Polish annual mercury emissions amount to approximately 10 Mg. Over 90% of mercury emissions in Poland originate from combustion of coal.

The purpose of this paper was to understand mercury behaviour during sub-bituminous coal and lignite combustion for flue gas purification in terms of reduction of emissions by active methods. The average mercury content in Polish sub-bituminous coal and lignite was 103.7 and 443.5 μg kg^−1^. The concentration of mercury in flue gases emitted into the atmosphere was 5.3 μg m^−3^ for sub-bituminous coal and 17.5 μg m^−3^ for lignite. The study analysed six low-cost sorbents with the average achieved efficiency of mercury removal from 30.6 to 92.9% for sub-bituminous coal and 22.8 to 80.3% for lignite combustion. Also, the effect of coke dust grain size was examined for mercury sorptive properties. The fine fraction of coke dust (CD) adsorbed within 243–277 μg Hg kg^−1^, while the largest fraction at only 95 μg Hg kg^−1^. The CD fraction < 0.063 mm removed almost 92% of mercury during coal combustion, so the concentration of mercury in flue gas decreased from 5.3 to 0.4 μg Hg m^−3^. The same fraction of CD had removed 93% of mercury from lignite flue gas by reducing the concentration of mercury in the flow from 17.6 to 1.2 μg Hg m^−3^. The publication also presents the impact of photochemical oxidation of mercury on the effectiveness of Hg vapour removal during combustion of lignite. After physical oxidation of Hg in the flue gas, its effectiveness has increased twofold.

## Introduction

Mercury is a highly toxic heavy metal with no physiological relevance for living organisms. Due to its adverse impact on health, global distribution, long atmospheric lifetime and propensity for deposition in the aquatic environment and in living tissue (Selin 2009), the US Environmental Protection Agency (US EPA) has classified mercury and its compounds as severe air quality threats (US EPA 1998). Global scale research has already confirmed the adverse health effects of mercury and fully justifies all current activities aimed at reduction of global mercury pollution (UNEP 2013). Especially, alarming are the results of research that enabled calculation of oceanic deposition of mercury at depths up to 1000 m, estimating the quantity at 64000 Mg of this element (Lamborg et al. 2014).

Such widespread presence of mercury in the environment originates from natural and anthropogenic sources (Pirrone et al. 2012; Kocman et al. 2013). It is estimated for natural mercury emissions to account for 5200 ± 2700 Mg per annum (Gustin et al. 2008; Pirrone et al. 2012; Kocman et al. 2013).

Global anthropogenic-derived emission of mercury is estimated at approximately 2000 Mg (AMAP/UNEP 2013), with the largest share of emissions attributed to artisanal mining and small-scale gold mining (37%), coal combustion (24%), mining, metallurgical industry and non-ferrous metal production (10%) and cement manufacture (9%). Average mercury emissions in the EU in 2010 amounted to 87.5 Mg. These emissions originated mostly from combustion processes (50%), cement manufacture (15%) and non-ferrous metal production (13%) (AMAP/UNEP 2013).

Poland and Germany are reported to have the highest annual mercury emissions in Europe. According to the National Centre for Emissions Management (KOBiZE) report for 2014, Poland is responsible for approximately 10 Mg of mercury pollution (KOBiZE 2015). This emission volume was attributed mostly to power plant coal combustion (54.3%), industrial combustion processes (29.4%), non-industrial combustion processes (10.5%) and metallurgy manufacturing processes (5.4%) (KOBiZE 2015). Collectively, over 90% of mercury emissions in Poland come from combustion of coal. In 2013, about 87% (National Report 2015) of electric energy and heat was generated from 35.325 million Mg of sub-bituminous coal and 61.798 million Mg of lignite (Burmistrz 2016; KOBiZE 2015).

The magnitude of coal combustion-derived mercury emissions into various parts of the environment depends mainly on content and chemical composition of combusted coal (especially chlorine, bromine, sulphur, iron, calcium), boiler type, temperature, flue gas constituents, fly ash properties (especially unburned carbon content) and flue gas cleanup technologies used (e.g. selective catalytic nitrous oxides reduction catalysts can also oxidise Hg^0^ to Hg^2+^) (Zhang et al. 2008; Wichliński et al. 2013; Ticknor et al. 2014; Dziok et al. 2015; Burmistrz et al. 2016).

The results of both industrial and pilot scale installations suggest that high concentration of bromine and chlorine have a positive impact on mercury reduction by Hg^2+^ into an intermediate form of Hg_(p)_ (Gale et al. 2008; Chmielniak et al. 2010). In other words, the higher the bromine and chlorine content in combusted coal, the more Hg^2+^ is formed, and consequently, the higher the mercury removal rate from a conventional cleanup system (Pavlish et al. 2003;, Wang et al. 2010; Burmistrz et al. 2016).

Studies in 84 power plants indicated that the distribution of various forms of mercury in total air emission of mercury is as follows: 87% Hg^0^, 5% Hg^2+^ and 8% Hg_(p)_ (Jensen et al. 2004; Burmistrz et al. 2016). Thus, mercury conversion from one form to another is essential in the selection of flue gas mercury removal technology.

Enhancing the effectiveness of the limitation of emissions of mercury to the atmosphere is made possible by increasing the share of oxidised mercury Hg^2+^ in the flue gas. In addition to chemical methods, resulting in complications in the APCD (Air Pollution Control Device) (Liu et al. 2014).), application of UV radiation is an effective way of oxidation of mercury. Ultraviolet radiation does not affect mercury contained in flue gas directly but causes oxygen molecules to break up into two radicals, which then react with metallic mercury, oxidising it to Hg^2+^. Oxygen radicals also react with oxygen molecules to form ozone, which (along with oxygen radicals) is the second potential factor contributing to oxidation of metallic mercury. Hg^0^ in the presence of UV radiation can also react with water vapour. The amount of oxidised mercury in flue gases depends on, among others, the wavelength of ultraviolet radiation and on the quantity of oxygen in the process flow (Liu et al. 2014).

There are several methods of mercury flue gas removal in combustion of solid fuels. They can be divided into primary (pre-combustion) methods, which include selective mining, coal enrichment and thermal coal treatment. Secondary methods (post-combustion) are further divided into passive and active methods. Passive methods are considered an efficient way of increasing oxidised mercury Hg^2+^ content in flue gas, which is captured much more efficiently by active methods. Passive methods, especially for combustion of lignites that are known for high concentrations of mercury and unfavourable chemical composition (low chlorine, bromine and iron with high calcium content) are ineffective, consequently resulting in mercury-contaminated flue gas (Bujny et al. 2012; Clack 2014). In such cases, additional technologies (active methods) must be applied to decrease emission of mercury to the atmosphere. Adsorption is the most efficient of these in terms of protection of the environment. It utilises mostly activated carbon, due to its high specific surface.

The estimated costs of injected activated carbon for 500 MWe boiler are around 3–4 USD/kW, while for lower power, these costs are higher and are around 8 USD/kW. The annual costs of activated carbon for 250 MW boiler range from 796,000 to 2 562,000 USD/year, depending on the type of ESP applied (Sloss 2008). According to recent data (Laurén 2016), the annual costs of untreated activated carbon injection were determined to be in the range of 20,225,000–420,674 USD/kgHg, depending on the plant size, emission limit, existing equipment and applied mercury removal technology. For 800 MW, power plant leads to operating costs even 955,056–3,820,225 USD/year (EPPSA Report 2015). Such costs are large and significantly restrict the large-scale application of this method.

The method is also problematic in terms of environmental protection, as production and regeneration processes have an adverse effect on the environment itself (Burmistrz 2016).

The purpose of this paper is to understand mercury behaviour during coal combustion and purification of flue gases in terms of emission reduction by active methods. Flue injection of dust-sized sorbents was the main technology of interest of the various active methods available. One of the main objectives of this study is to recommend a low-cost organic sorbent such as lignite dust, coke dust and tire char to efficiently substitute expensive dust-sized activated carbon. The study covered combustion of sub-bituminous coal and lignite from Polish mines and fields. The experiment was conducted at temperatures reflecting conditions inside a flue gas purification installation. UV radiation of various wavelengths was also examined to analyse the impact on the effectiveness of removing mercury from coal and lignite flue gas.

## Material and methods

### Applied sorptive materials

Sorption studies of mercury in flue gases consisted of four types of organic sorbents in six different forms. Their characteristics are shown in Table 1.

**Table 1:** Description of organic sorbents used in the study

No.	Sorbent	Symbol	Material origin
1	Activated carbon	AC	Commercial activated carbon, dedicated i.a. for gas-phase mercury removal. Formed in the process of coal carbonisation and subsequent thermal activation of the obtained structure.
2	Coke dust	CD	By-product of large scale coke production. The dust is obtained during coke dry-cooling process, hauling and sorting.
3	Lignite dust	LD	By-product of lignite extraction, preparation and transport. Lignite dust is obtained from raw lignite treated in various processes, from coal fragmentation, drying and sorting to milling.
4	Lignite dust char	LDC	Product of pyrolysis of lignite dust at 1123 K
5	Rubber char 1	RC	Solid product (char) of car tyre pyrolysis at 1123 K. The material was derived from industrial installations in which intact tires are subjected to high temperatures.
6	Granulated rubber char 2	RGC	Solid product (char) of granulated car tyre (2–4 mm particle size) pyrolysis at 1123 K

### Sorbent analysis

The scope of sorbent analysis listed in the “Applied sorptive materials” section includes:

(i) Proximate and ultimate analysis in accordance with the ISO standard (ISO 17246:^2010^, ISO 17247:^2013^),
(ii) Determination of chlorine content evaluated as chlorine anion content in water solution using a direct reading spectrophotometer (DR/2000 HACH). A sample was combusted in AC-350 bomb calorimeter (LECO) with Eschka mixture—in accordance with the ISO standard (ISO 587:^2000^),
(iii) Mercury content analysis by absorptive atomic spectrometry with cold vapour (CV-AAS: DMA-80 Direct mercury analyser; Milestone Connect),
(iv) Analysis of particle size of analysed sorbents by ISO standard (ISO 728:^1995^),
(v) The porous texture of all samples was analysed using nitrogen adsorption/desorption at 77 K using Autosorb®-1-C (Quantachrome Instruments, USA). Before measurements were made, all samples were degassed at 473 K for 12 h under vacuum. Interpretation of textural properties: specific surface area by Brunauer-Emmett-Teller (BET), volume of micropore calculated by Dubinin-Raduszkiewicz method, volume of mesopore calculated by Barrett, Joyner and Halenda method (BJH) and total volume of pores was carried out in accordance with the recommendations of the following standards: (NIST 2006, ISO 9277: 2010, ISO 15901-2: 2006, ISO 15901-3: 2007).

Values of these parameters were determined for the air-dried basis of the sample.

### Flue gas mercury adsorption during coal combustion

**Fig. 1:**
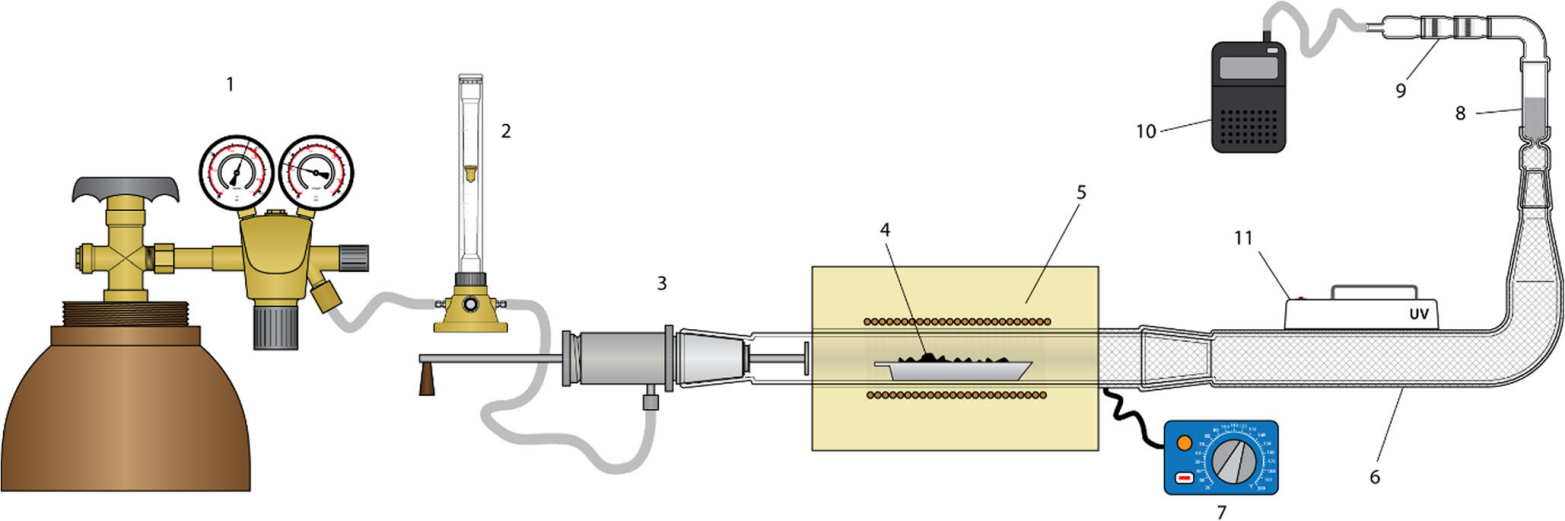
Test stand schematic. 1 source of oxidizer, 2 rotameter, 3 oxidizer supply system, 4 coal sample, 5 tube furnace, 6 heated flue gas stream, 7 temperature regulator, 8 glass vessel with sorbent, 9 double sorbent trap, 10 pump, 11 UV analytical lamp (optional)

Figure 1 shows a schematic of a test stand for measuring mercury sorption from flue gases generated by combustion of solid fuels. It consists of a temperature-regulated tube furnace, quartz combustion chamber, gas cylinder, flow meter and sorbent placed in a container. The temperature of flue gases can be controlled between the quartz tube outlet and combustion pipe. The fuel sample is combusted in the flow of air, and flue gases are directed through the sorbent container. Subsequently, the amount of adsorbed mercury is measured. The analysis is performed in defined, controlled conditions which include combustion temperature and time, temperature of flue gases passing through the sorbent and flow rate. The fuel sample is inserted in a small ceramic container, which is gradually transferred into the area of highest temperature. The sorbent container is located inside a heating element which stabilises the sorbent temperature during measurement. This enables simulation of industrial conditions as well as analysis of mercury sorption at different temperatures.

A sorbent trap is placed after an organic sorbent. The trap measures the mass of mercury emitted from the combustion gases into the atmosphere. Before the procedure, mercury content is measured in both fuel and sorbent using the methods described in the “Sorbent analysis” section. After the procedure, mercury content is analysed in ash, sorbent and in the sorbent trap.

For the combustion process, sub-bituminous and lignite coal was used. The sub-bituminous and lignite coal underwent ultimate and proximate analysis, with the additional steps of mercury and chlorine determination in accordance with the methodology described in the “Sorbent analysis” section. In addition, ashes of the coal samples were examined for Fe_2_O_3_ and CaO content with Energydispersive X-ray fluorescence spectrometer (EDXRF): Panalytical Epsilon 3^XLE^.

Sample preparation was applied in accordance with respective ISO standards (ISO 5069-2:^1983^). The characteristics of combusted coals are shown in Tables 5 and 6.

The experimental conditions were the same in all instances (Table 2).

**Table 2:** Experimental conditions

Parameter	Description
Combustion temperature (K)	1123
Flue gas flow rate (cm^3^ s^−1^)	2.0
Combustion time (s)	900
Mass of sorbent (g)	0.6
Volume of flue gases (cm^3^)	800

The impact of photochemical oxidation on flue gas mercury adsorption was also included in the study. Analytical lamp (UV-240 MERAZET/254 nm + 366 nm/2 × 6 W) was used as the radiation source and was installed right after the combustion area and before the sorbent container (Fig. 1). A wavelength of 254 nm at 15 cm–500 μW cm^−3^ was used in the study.

### Methodology of mercury adsorption

The following parameters were determined during the mercury flue gas adsorption process: (i) mass of combusted coal and mercury content (m_c_, C_0_), (ii) weight of tested sorbent and its mercury content before and after sorption (m_a_, C′_Hg_, C″_Hg_), (iii) mercury content in the ash remaining after combustion of coal (C_ash_), (iv) mercury content in the trap before and after the experiment (C′_gas_, C″_gas_). On the basis of obtained data and initial measurements (Table 2), flue gas mercury concentration before and after adsorption and the following parameters were calculated:

1 $$ \left(\mathrm{i}\right)\kern0.5em \mathrm{Quantity}\ \mathrm{of}\ \mathrm{adsorbed}\ \mathrm{mercury}\ \left[ ug\cdot {kg}^{\hbox{-} 1}\right],{A}_{Hg}={C}_{Hg}^{"}-{C}_{Hg}^{\hbox{'}} $$

2 $$ \left(\mathrm{ii}\right)\kern0.5em \mathrm{Efficiency}\ \mathrm{of}\ \mathrm{mercury}\ \mathrm{removal}\ \left[\%\right],\mathrm{MR}=\frac{{\mathrm{A}}_{\mathrm{Hg}}}{{\mathrm{C}}_0-{\mathrm{C}}_{\mathrm{ash}}}\cdot 100\% $$

To check the reliability of the tests performed, the balance of mercury in the laboratory installation was calculated for each experiment in accordance with the following model:

3 $$ \left(\mathrm{iii}\right)\kern0.5em {m}_c\cdot {C}_0-{m}_{ash}\cdot {C}_{ash}={m}_a\cdot \left({C}_{Hg}^{"}-{C}_{Hg}^{\hbox{'}}\right)+{m}_{trap}\cdot \left({C}_{gas}^{"}-{C}_{gas}^{\hbox{'}}\right) $$

where *m*_ash_—mass of ash from coal combustion, *m*_trap_—mass of active carbon in gas trap 9 (see Fig. 1).

## Results and discussion

### Mercury analysis in coal, ash and sorbents

Validation studies confirmed that the CV-AAS method using mercury analyser MA-3000 (Nippon Instruments Corporation) is accurate for coal samples with mercury content ranging from 25 to 600 μg kg^−1^ in air dried basis. The detection limit is 0.07 ng, and the quantification limit is 0.20 ng. The method is highly linear (*r* = 0.998), and uncertainty of results at 95% confidence level ranges from 3 to 10%, depending on measurement. The CV-ASS method has acceptable repeatability and reproducibility in the whole measurement range.

In other materials, including ashes and organic sorbents, the CV-AAS method was accurate for mercury content ranging from 5 to 1200 μg kg^−1^ with relative uncertainty from 2 to 20%. The highest values of relative uncertainty were noted for ash samples and AC, CD and LDC samples with mercury content below 10 μg kg^−1^.

### Sorbent properties

LD contained 68.3 μg Hg kg^−1^, and its char (LDC) only 3.3 μg kg^−1^. Likewise, CD and AC, which are products of the coal carbonisation process, contained relatively small quantities of mercury: 10.5 and 5.4 μg kg^−1^, respectively (Table 3). CD, LDC and AC are materials obtained in the carbonisation process. Therefore, they also contain minimal quantities of volatiles: approximately 3 wt% for CD and LDC and approximately 15% for AC while LD contained close to 50%. Commercial activated carbon (AC) had more than 2.5 times higher ash content than other examined sorbents. Sulphur content in AC of 2.11 wt% was more than double than in the other sorbents. RC and RGC have high mercury content, at 158 and 73 μg kg^−1^, high sulphur content (2.26 and 2.45 wt%) and ash (19.4 and 23.2%), for RC and RGC, respectively.

**Table 3:** Properties of analysed sorbents

Sorbent (see Table 1)	M_ad_	V_ad_	A_ad_	Cl_ad_	C_ad_	H_ad_	S_ad_	Hg_ad_
	(wt%)							(μg kg^−1^)
AC	9.2	15.09	26.2	–	59.5	1.45	2.11	5.4
CD	0.4	3.19	9.8	0.20	85.0	0.16	0.59	10.5
LD	8.0	49.39	5.2	0.05	58.4	5.25	0,54	68.3
LDC	0.1	3.35	9.6	0.03	87.9	0.93	0.92	3.3
RC	0.9	–	23.2	–	72.8	0.89	2.26	158.1
RGC	0.8	–	19.4	–	76.2	0.80	2.45	73.0

*M*_*ad*_ moisture in the air-dried basis, *V*_*ad*_ volatile matter in the air-dried basis, *A*_*ad*_ ash in an air-dried basis, *Cl*_*ad*_ chlorine in the air-dried basis, *C*_*ad*_ carbon in the air-dried basis, *H*_*ad*_ hydrogen in the air-dried basis, *S*_*ad*_ sulphur in the air-dried basis, *Hg*_*ad*_ mercury in the air-dried basis

Coke and lignite dust are macroporous materials with a moderately developed mesoporous and poor microporous structure (Table 4). Specific surface (S_BET_) of CD, LD and LDC amounts to several dozen square meters per·gram, but in AC this parameter is 670.5 m^2^ g^−1^. Due to these differences in structure, the equilibrium sorptive capacity of AC is more than 50 times higher for mercury than in those of the three other sorbents (Table 4). The specific surface area of rubber waste chars was approximately 70 m^2^ g^−1^.

**Table 4:** Parameters of porous structure for analysed sorbents, based on nitrogen vapour sorption/desorption isotherms measured at 77 K

Sample	S_BET_ (m^2^/g)	V_DR_ (cm^3^/g)	V_BJH_ (cm^3^/g)	V_total_ (cm^3^/g)	SC_Hg_ (mg g^−1^)
AC	670.5	0.307	0.055	–	24.052
CD	24.3	0.006	0.009	0.018	0.110–0.129
LD	3.5	0.001	0.025	0.054	0.279
LDC	4.5	0.001	0.032	0.065	–
RC	70.3	0.025	0.380	0.396	–
RGC	74.7	0.023	0.171	0.239	–

*SC*_*Hg*_ sorption capacity of mercury, as milligrams per gram, *S*_*BET*_ specific surface based on Brunauer-Emmet-Teller method, *V*_*DR*_ micropore volume based on Dubinin-Raduszkiewicz method, *V*_*BJH*_ mesopore volume based on Barret-Joyner-Halenda method, *V*_*total*_ total pore volume

### Properties of sub-bituminous coal and lignite

Sub-bituminous coal contained an average of 103.7 μg kg^−1^ of mercury, while average mercury content in lignite was 443.5 μg kg^−1^ (Table 5). The average content of chlorine, which is a supporting factor in oxidation of mercury from Hg^0^ to Hg^2+^ in analysed sub-bituminous coal was equal to 0.3%, while lignite contained nearly seven times less chlorine. The average sulphur content for coal and lignite was 0.6 and 1.8 wt%. Lignite contained more than three times more ash, twice as many volatiles and ten times more moisture than sub-bituminous coal.

**Table 5:** Characterisation of coals used in the experiment

Coal	M_ad_	V_ad_	A_ad_	Cl_ad_	C_ad_	H_ad_	S_ad_	Hg_ad_
	(wt%)							(μg kg^−1^)
Sub-bituminous coal	1.13	22.74	7.6	0.33	83.8	4.65	0.58	103.7
Lignite	12.9	41.20	23.7	0.05	43.5	4.90	1.80	443.5

There was a noticeable amount of iron in lignite (18.5 wt%.), a factor proven to catalyse oxidation of mercury, whereas the iron content in sub-bituminous coal was equal to 4.8 wt% (Table 6). The content of calcium, which competes with mercury in binding of chlorine accounted for 2.3 wt% for sub-bituminous coal and 14.3 wt% for lignite (Table 6).

**Table 6:** Fe and Cu content in the ash of sub-bituminous coal and lignite

Coal	Fe	Ca
	(wt%)	
Sub-bituminous coal	4.79	2.26
Lignite	18.49	14.34

### Assessment of the effectiveness of mercury sorption during combustion of lignite and sub-bituminous coals

Table 7 shows mercury sorption values on tested sorbents and effectiveness of mercury removal from flue gas. Commercial activated carbon, currently used in active flue gas mercury removal methods, was the most efficient, removing the mercury almost entirely. CD also shows high mercury sorption efficiency from both sub-bituminous coal and lignite—at 80 and 63%, respectively. Observations have shown lignite dust to be the worst sorptive material during coal combustion, decreasing the concentration of mercury in flue gas by only 30%, and by 23% in the combustion of lignite. LDC was more efficient in mercury removal than LD.

**Table 7:** Effectiveness of organic sorbents for mercury sorption

Coal	Hg_coal_	Sorbent	A_Hg_	MR
	(μg kg^−1^)		(μg kg^−1^)	(%)
Sub-bituminous coal	103.7	AC	96.3	92.9
		CD	83.2	80.3
		LD	31.7	30.6
		LDC	91.3	88.1
		RC	81.6	78.7
		RGC	84.3	81.3
Lignite	443.5	AC	356.1	80.3
		CD	277.0	62.5
		LD	101.0	22.8
		LDC	221.8	50.1
		RC	374.7	84.5
		RGC	389.8	86.6

**Fig. 2:**
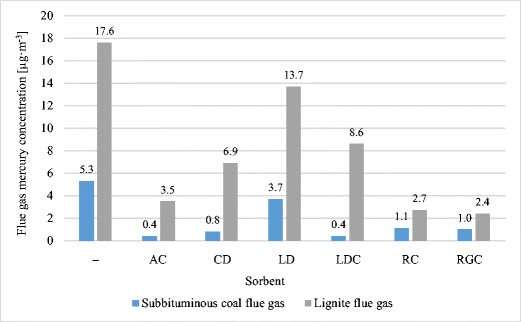
Concentration of mercury in raw flue gas compared to cleaned flue gas emitted to the atmosphere

In the combustion process of lignite with mercury content of 443.5 μg kg^−1^, raw flue gas concentration of mercury amounted to 17.6 μg m^−3^—more than three times higher than the concentration of mercury in flue gas from combustion of sub-bituminous coals (Fig. 2).

The high concentration of mercury in flue gas from combustion of lignite is due to its high initial mercury content and low chlorine content (0.05 wt%, for sub-bituminous coal—3.3 wt%).

Most of the mercury from the combustion of sub-bituminous coal and lignite was removed by AC sorbent. After completing the process of coal combustion, the mercury content in sorbent was 96.3 and 356.1 μg kg^−1^, respectively for combustion of sub-bituminous coal and lignite (Table 7). The concentration of mercury in flue gas from coal and lignite were reduced to a value of 0.4 and 3.5 μg kg^−1^ (see Fig. 2).

A_Hg_ for CD was 83.2 and 277.0 μg Hg kg^−1^, which enabled a reduction of mercury concentration in flue gas to a value of 0.8 μg m^−3^ for coal and 6.9 μg m^−3^ for lignite. A comparable value of A_Hg_ was obtained for LDC, as mercury content determined in the flue did not exceed 91.3 for sub-bituminous coal and 221.8 μg kg^−1^ for lignite. The result was a reduction in the concentration of mercury in flue gas to 0.4 and 8.6 μg m^−3^.

Application of LD sorbent resulted in poor mercury removal. A_Hg_ for the combustion of coal was 31.7 and 101.0 μg kg^−1^ for lignite, resulting in 1.35 times the reduction of atmospheric mercury emission (see Table 7).

RC and RGC sorbents provided better mercury removal during combustion of lignite, where mercury flue gas concentration after using RC and RGC was 2.7 and 2.4 μg kg^−1^, respectively (see Fig. 2). Each of the sorbents used adsorbed more mercury from flue gases from lignite combustion than from sub-bituminous coal. As already mentioned, such adsorption was the consequence of mercury concentration that was three times higher in lignite than in sub-bituminous coal.

The best sorbent was AC, removing 92.9% of mercury from sub-bituminous coal and 80.3% of mercury from lignite flue gas (Table 7). The poorest results were obtained with LD sorbent, capturing up to 30.6 and 22.8% of the mercury in an equivalent experiment. Results comparable to MR were obtained for CD and LDC. In sub-bituminous coal flue gas, CD and LDC removed 80.3 and 88.1% of mercury. In flue gas from lignite, this parameter reached approximately 62.5 and 50.1%.

All tested sorbents were more effective in removing mercury from flue gas from sub-bituminous coal (30.6 to 92.9%) than from lignite (MR from 22.8 to 80.3%). This was most likely due to a larger share of the more easily adsorbed Hg^2+^ during coal combustion, whereas lignite flue gas had a much greater content of the difficult to adsorb Hg^0^.

A greater share of Hg^2+^ in flue gas from sub-bituminous coal was the consequence of a greater content of chlorine and lower amount of calcium in coal than in lignite. This resulted in different mercury oxidation potentials during both combustion processes.

**Fig. 3:**
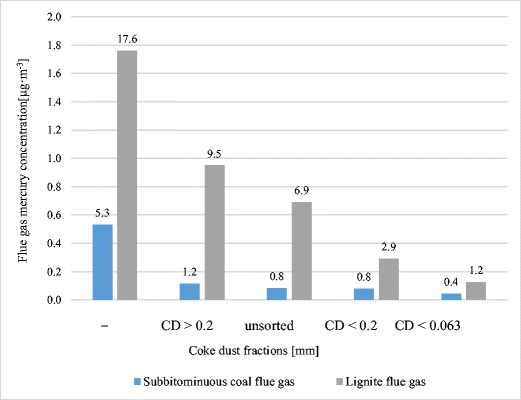
Effect of sorbent particle size on flue gas mercury removal

RC and RGC proved to be highly capable of mercury sorption from coal combustion flue gas (79%, 81%), showing even better efficiency in the removal of mercury during combustion of lignite (85 and 87%). The concentration of mercury in emitted flue gas, with RC and RGC sorbents decreased by more than 80% (see Fig. 3).

### Effect of sorbent particle size on mercury sorption ability

To investigate the effect of particle size on the effectiveness of mercury removal, sorbent CD was screened to form the following fractions: (i) > 0.2 mm, (ii) < 0.2 mm, and (iii) < 0.063 mm. The properties of individual grain fractions are shown in Table 8. The mercury content in individual size fractions differed only slightly. The finer grains contained slightly more ash and volatiles, and less carbon. The < 0.063-mm fraction contained 14.8 μg Hg kg^−1^, while the > 0.2-mm fraction—11.2 μg kg^−1^.

**Table 8:** Properties of selected fractions of CD sorbent particles

Sorbent	M_ad_	V_ad_	A_ad_	C_ad_	S_ad_	Hg_ad_
	(wt%)					(μg kg^−1^)
CD > 0.2 mm	1.3	5.1	12.0	79.8	0.65	11.2
CD < 0.2 mm	2.6	6.7	17.7	75.1	1.02	12.1
CD < 0.063 mm	1.6	7.1	17.8	73.4	1.05	14.8

Significant differences in sulphur content in individual fractions were also observed: from 0.65% in the coarsest to more than 1% in the finest.

The finer the CD fraction, the better the mercury adsorption (A_Hg_) and effectiveness of removal of mercury from flue gas (Table 9). A particularly strong impact of particle size on mercury adsorption was found in sorption of flue gas from lignite. The finest fraction of CD adsorbed 243–277 μg Hg kg^−1^ CD, while the coarsest fraction adsorbed only 95 μg Hg kg^−1^.

**Table 9:** Effect of sorbent particle size on mercury sorption ability and flue gas mercury removal

Coal	Hg_coal_	Fraction	A_Hg_	MR
	(μg kg^−1^)		(μg kg^−1^)	(%)
Sub-bituminous coal	103.7	CD > 0.2	78.8	76.0
		CD < 0.2	83.2	85.2
		CD < 0.063	88.3	91.9
Lignite	443.5	CD > 0.2	95.2	54.9
		CD < 0.2	243.0	83.2
		CD < 0.063	277.0	93.1

Observed regularity was reported by other researchers for other sorbents, i.e. activated carbons (McKay et al. 2015). Authors have demonstrated that a stepwise decrease of activated carbon particle size produced resulted in an increase in mercury adsorption (Kadirvelu et al. 2004).

The < 0.063-mm fraction of CD removed almost 92% of mercury from the flue gas of sub-bituminous coal, reducing mercury concentration from 5.3 to 0.4 μg Hg m^−3^. The same fraction of CD removed 93% of mercury from lignite flue gas, reducing the concentration of mercury in flue gas from 17.6 to 1.2 μg Hg m^−3^ (Fig. 3).

### Influence of photochemical oxidation on gas phase mercury

**Fig. 4:**
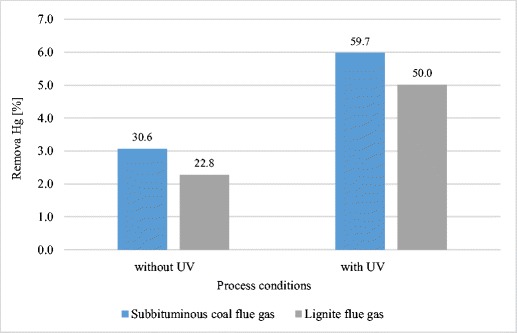
Impact of photochemical oxidation on sorption of mercury on LD sorbent

**Fig. 5:**
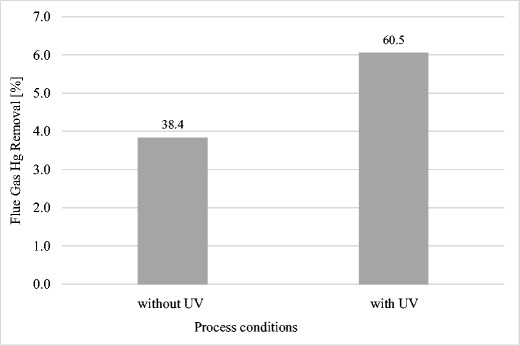
Impact of photochemical oxidation on sorption of mercury on LDC sorbent

After physical oxidation of mercury in the flue gas, the sorption of mercury from the flue gas from sub-bituminous coal and lignite was conducted with the use of LD and LDC (only for lignite emissions; however, because LDC reduced the concentration of mercury in flue gas of sub-bituminous coal to 0.4 μg kg^−1^ without photochemical oxidation of Hg, so it was not used in this experiment for this fuel). The results obtained were better than in trials carried out without UV radiation (see Fig. 4 and Fig. 5).

The method of photochemical oxidation of Hg^0^ to Hg^2+^, most likely by the mechanism described in Liu et al. (2014) allowed for 1.5- to 2-fold increase in the quantity of mercury adsorbed. LD during combustion of coal and lignite decreased the concentration of mercury in flue gas by 30 and 23%, respectively. After physical oxidation of Hg in the flue gas, this effectiveness increased to 60 and 50% (Fig. 4). A similar effect was obtained for LDC sorbent—after photochemical oxidation of mercury, the efficiency of mercury removal from flue gas from the combustion of sub-bituminous coal increased from 40 to 60%. (Fig. 5).

## Conclusions

The results of studies of mercury adsorption from coal and lignite flue gas using six organic sorbents (AC, CD, LD, LDC, RC and RGC) allow for the formulation of the following conclusions.

Greatest mercury removal from flue gas from coal was achieved with AC (92.9%), followed by LDC (88.1%), RGC (81.3%), CD (80.3%) and RC (78.7%). In removal of mercury from lignite flue gas, the greatest effectiveness was achieved with RGC (86.6%), followed by RC (84.5%) and AC (80.3%). AC, CD, LD and LDC were more effective in removing mercury from flue gas from sub-bituminous coal than from lignite, while the RGC and RC were more effective in removing mercury from lignite flue gas than from sub-bituminous coal flue gas.

The finer the CD grain size, the higher the effectiveness of mercury removal from flue gas. For coal flue gas, the < 0.063-mm fraction captured 91.9% as compared to 76.0% removed by the > 0.2 mm-fraction. For lignite flue gas, the < 0.063-mm fraction removed 93.1% of mercury as compared to 54.9% for the > 0.2-mm fraction.

The use of < 0.063-mm fraction of CD enabled a mercury decrease in flue gas: (i) in sub-bituminous coal, from 5.3 to 0.4 μg m^−3^ and (ii) in lignite, from 17.6 to 1.2 μg m^−3^.

The use of UV radiation has increased the effectiveness of adsorptive properties of LD. Mercury removal from lignite flue gas has more than doubled, from 22.8 to 50.0%. The effect of UV radiation on sub-bituminous coal flue gas was lower, increasing the effectiveness of mercury removal from 30.6 to 59.7%.

## References

[CR1] AMAP/UNEP, Technical Background Report for the Global Mercury Assessment (2013) Arctic Monitoring and Assessment Programme, Oslo, Norway/UNEP chemicals branch, Geneva

[CR2] Bujny M, Burmistrz P, Gruszka S, Janicki W, Kogut K, Strugała A (2012). Removing mercury from flue gases. A demo plant based on injecting dusty sorbents (in Polish). Energy Policy J.

[CR3] Burmistrz P, Kogut K, Marczak M, Zwoździak J (2016). Lignites and subbituminous coals combustion in Polish power plants as a source of anthropogenic mercury emission. Fuel Process Technol.

[CR4] Burmistrz P (2016) Fuel technology challenges and opportunities. Kraków, 104–115

[CR5] Chmielniak T, Głód K, Misztal E, Kopczyński M (2010). Mercury emission from coal-fired power plants. Przemysł Chemiczny.

[CR6] Clack H (2014) Methods for reducing mercury emission from coal combustion. Mercury as a coal combustion pollutant:105–120

[CR7] Dziok T, Strugała A, Rozwadowski A, Macherzyński M (2015). Studies of the correlation between mercury content and the content of various forms of sulfur in Polish hard coals. Fuel.

[CR8] EPPSA Report (2015) Mercury removal guideline for assessment and design recommendations. European Power Plant Suppliers Association

[CR9] Gale T, Lani B, Offen G (2008). Mechanisms governing the fate of mercury in coal-fired power systems. Fuel Process Technol.

[CR10] Gustin MS, Lindberg SE, Weisberg PJ (2008). An update on the natural sources and sinks of atmospheric mercury. Appl Geochem.

[CR11] ISO 5069-2: 1983 Brown coals and lignites—part 2: sample preparation for determination of moisture content and for general analysis

[CR12] ISO 728:1995—Size analysis by sieving

[CR13] ISO 587:2000—Solid mineral fuels—determination of chlorine using Eschka mixture

[CR14] ISO 15901-2: 2006—Pore size distribution and porosity of solid materials by mercury porosimetry and gas adsorption—part 2: analysis of mesopores and macropores by gas adsorption

[CR15] ISO 15901-3: 2007—Pore size distribution and porosity of solid materials by mercury porosimetry and gas adsorption—part 3: analysis of micropores by gas adsorption

[CR16] ISO 9277: 2010—Determination of the specific surface area of solids by gas adsorption—BET method

[CR17] ISO 17246:2010, Coal—proximate analysis

[CR18] ISO 17247:2013, Coal—ultimate analysis

[CR19] Jensen RR, Karki S, Salehfar H (2004). Artificial neural network-based estimation of mercury speciation in combustion flue gases. Fuel Process Technol.

[CR20] Kadirvelu K, Kavipriya M, Karthika C, Vennilamani N, Pattabhi S (2004). Mercury (II) adsorption by activated carbon made from sago waste. Carbon. N Y.

[CR21] KOBiZE (National Centre for Emissions Management) (2015), Poland’s Informative Inventory Report

[CR22] Kocman D, Horvat M, Pirrone N, Cinnirella S (2013). Contribution of contaminated sites to the global mercury budget. Environ Res.

[CR23] Lamborg CH, Hammerschmidt CR, Bowman KL, Swarr GJ, Munson KM, Ohnemus DC, Lam PJ, Heimbürger LE, Rijkenberg MJA, Saito MA (2014). A global ocean inventory of anthropogenic mercury based on water column measurements. Nature.

[CR24] Laurén V (2016) Technical and economic study on mercury emission control technologies for combustion power plants. Available: https://aaltodoc.aalto.fi/bitstream/handle/123456789/23988/master_Laur%C3%A9n_Ville_2016.pdf?sequence=1&isAllowed=y

[CR25] Liu J, Zhang J, Yin Y (2014). Study on absorption of elemental mercury from flue gas by UV/H_2_O_2_: process parameters and reaction mechanism. Chem Eng J.

[CR26] McKay G, Hadi P, Hui CW, Sze Ki Lin C, To M H (2015). Aqueous mercury adsorption by activated carbons. Water Res.

[CR27] NIST 2006 —Porosity and specific surface area measurements for solid materials

[CR28] Pavlish JH, Sondreal EA, Mann MD, Olson ES, Galbreath KC, Laudal DL, Benson SA (2003). Status review of mercury control options for coal-fired power plants. Fuel Process Technol.

[CR29] Pirrone N, Cinnirella S, Feng X, Finkelman RB, Friedli HR, Leaner J, Mason R, Mukherjee AB (2012). Report on activity of president of energy regulator office in 2011. Warszawa.

[CR30] Selin NE (2009). Global biogeochemical cycling of mercury: a review. Annu Rev Environ Resource.

[CR31] Sloss L (2008) Economics of mercury control. CCC:134

[CR32] Ticknor JL, Hsu-Kim H, Deshusses MA (2014). A robust framework to predict mercury speciation in combustion flue gases. J Hazard Mater.

[CR33] US EPA (1998). A study of hazardous air pollutant emissions from electric utility steam generating units: final report to congress; EPA-453/R-98-004a, US EPA Office of Air Quality Planning and Standards.

[CR34] UNEP (2013) Mercury. Time to Act (Geneve)

[CR35] Wang SXL, Zhang L, Li GH, Wu Y, Hao J, Pirrone N, Sprovieri F, Ancone MP (2010). Mercury emission and speciation of coal-fired power plants in China. Atmos Chem Phys.

[CR36] Wichliński M, Kobyłecki R, Bis Z (2013). The investigation of mercury contents in Polish coal samples. Arch Environ Protec.

[CR37] Zhang L, Zhuo Y, Chen L, Xu X, Chen C (2008). Mercury emission from six coal-fired power plants in China. Fuel Process Technol.

